# Using AST-platelet ratio index and fibrosis 4 index for detecting chronic hepatitis C in a large-scale community screening

**DOI:** 10.1371/journal.pone.0222196

**Published:** 2019-10-22

**Authors:** Yuan-Hung Kuo, Kwong-Ming Kee, Nien-Tzu Hsu, Jing-Houng Wang, Chang-Chun Hsiao, Yi Chen, Sheng-Nan Lu

**Affiliations:** 1 Division of Hepatogastroenterology, Department of Internal Medicine, Kaohsiung Chang Gung Memorial Hospital and Chang Gung University College of Medicine, Kaohsiung, Taiwan; 2 Graduate Institute of Clinical Medical Sciences, College of Medicine, Chang Gung University, Taoyuan, Taiwan; 3 Biostatistics and Bioinformatics Center of Kaohsiung Chang Gung Memorial Hospital, Kaohsiung, Taiwan; 4 Center for Shockwave Medicine and Tissue Engineering, Kaohsiung Chang Gung Memorial Hospital, Kaohsiung, Taiwan; 5 Public Health Bureau, Tainan City Government, Tainan, Taiwan; 6 Division of Hepatogastroenterology, Department of Internal Medicine, Chiayi Chang Gung Memorial Hospital, Chiayi, Taiwan; Kaohsiung Medical University, TAIWAN

## Abstract

**Background:**

Aspartate transaminase-platelet ratio index (APRI) and fibrosis 4 (FIB-4) are two non-invasive indexes to predict liver fibrosis in liver disease. This study was to use APRI and FIB-4 to detect chronic virus hepatitis in community screenings.

**Methods:**

From 2004 to 2013, a series of community-based health screenings for residents aged 40 and older were held in Tainan city. APRI and FIB-4 of each participant were calculated and their association further analyzed with hepatitis status.

**Results:**

We enrolled 180359 participants including 18726 (10.4%) hepatitis B virus (HBV), 13428 (7.4%) hepatitis C virus (HCV), 1337 (0.7%) HBV plus HCV and 146868 (81.5%) Non-HBV Non-HCV. The prevalence of chronic HCV increased with the elevation of APRI cut-offs or FIB-4 cut-offs (13.9%, 28.1%, 38.8%, 45.2%, to 49.9% in APRI≥0.3, 0.5, 0.7, 0.9,1.1, p<0.001 for the linear trend; or 15.8%, 26.4%, 34.4% to 39.7% in FIB-4≥1.75, 2.75, 3.5, 4.25, p<0.001). At the township level, APRI≥ 0.7 and FIB-4≥ 3.5 were highly correlated with HCV infection (r = 0.95, p<0.001 in APRI and r = 0.809, p<0.001 in FIB-4) and hepatocellular carcinoma (HCC) development (r = 0.894, p<0.001 in APRI and r = 0.804, p<0.001 in FIB-4), but not correlated with HBV infection.

**Conclusions:**

Community screenings derived APRI or FIB-4 can identify patient subsets with increased of underlying HCV infection and risk of incident HCC.

## Introduction

Globally, there are more than 350 million persons infected with chronic hepatitis B virus (HBV) [1]. It is particularly endemic in Taiwan, where HBV infection is usually acquired perinatally or in early childhood [2]. With the offer of HBV vaccine for the newborn in Taiwan since 1984, the annual incidence of HBV infection is decreasing [3]. However, those subjects with chronic hepatitis B (CHB) infection are still at an increased risk of developing progressive liver disease like liver cirrhosis (LC), or hepatocellular carcinoma (HCC), and liver-related motality [4]. Regarding hepatitis C virus (HCV), chronic hepatitis C (CHC) infection is the second leading cause of LC and HCC [5]. In Taiwan, the HCV prevalence is approximately 4.4% and nearly one million people are infected [6].

In the recent decade, new potent anti-viral drugs have improved the severity of liver disease and reduced its related mortality rate, no matter in HBV or HCV infection [7–9]. Especially for the new generation of direct-acting antiviral agents (DAA), the success rate of HCV eradication could even reach or go beyond 95% in naive and non-cirrhotic patients [10,11]. Since current anti-viral drugs are so effective in the control of viral load and the improvement of liver disease, how to identify and treat patients with HBV or HCV infection economically in endemic areas is crucial to for public health.

Every year, many community-based health screenings are conducted for different target diseases by the county or city public health bureaus in Taiwan. In general, serum items of examinations usually include biochemical tests and complete blood cell counts. Unless for a special hepatitis screening, virus markers such as hepatitis B virus surface antigen (HBsAg) and anti-HCV antibody (anti-HCV) are not tested under consideration of cost-savings. Even in Taiwan, in such HBV and HCV endemic areas, nationwide hepatitis screening is still difficult to perform. Aspartate transaminase (AST)-platelet ratio index (APRI) and fibrosis 4 (FIB-4) are two non-invasive indexes that are used to predict liver fibrosis with an acceptable diagnostic accuracy in different liver diseases [12–14]. Since the tests used to calculate APRI and FIB-4 are usually included in general health screenings in Taiwan, we aimed to describe differences in the prevalence of HCV and HBV infection based on increasing thresholds of APRI and FIB-4 performed in population-based screening.

## Methods

### Study subjects

Tainan County is located in southern Taiwan with approximately 1.1 million residents in 31 townships. From 2004 to 2013, a series of county-wide comprehensive community health examinations were conducted by the Public Health Bureau of Tainan County for residents older than 40 years of age [15–17]. Residents aged 40 years and above could participate in this series of screenings once every three years, whereas those above 65 years of age could participate every year. All participants underwent anthropometric measurements and blood tests included HBsAg, anti-HCV, biochemical tests, complete blood cell counts and alpha-fetoprotein (AFP). According to HBsAg and anti-HCV, status of hepatitis viral infection was divided into hepatitis B virus (HBV), HCV, B+C and non-HBV non-HCV (NBNC). APRI index was calculated using the following formula: (AST (IU/L) /upper-limit of normal)/ platelet count (10^9^ /L) x 100). Upper-limit of normal AST used for calculation was 33 IU/L [18]. While FIB-4 index was calculated as follow: (age (years) x AST (IU/L) / platelet count (10^9^ /L) x square root of Alanine transaminase (ALT) (IU/L)) [19]. The upper-limit of normal ALT was 40 IU/L. When participants had multiple visits, data from the first visit was used for the analysis. This study was approved by the Institutional Review Board of our institute: Kaohsiung Chang Gung Memorial Hospital. Besides, this study was also conducted according to the principles expressed in the Declaration of Helsinki.

### Statistical analysis

Average values were expressed as mean±SD. Correlation analysis was used to express the association of APRI / FIB-4 and the prevalence of HBV infection or HCV infection as well as the decade incidence of HCC in townships of Tainan. A probability lower than 0.05 level was defined as statistically significant. Statistical analysis was performed using SPSS 15.

## Results

### Baseline characters of enrolled subjects

Atotal of 180,359 participants including 73,556 (40.8%) male and 106,803 (59.2%) female participants with a mean age of 58.5±11.6 years were enrolled (Table 1). There were 18,726 (10.4%) HBV, 13,428 (7.4%) HCV, 1,337 (0.7%) B+C and 146,868 (81.5%) NBNC. In the status of liver inflammation such as AST and ALT or liver fibrosis such as APRI and FIB-4, those subjects with chronic virus hepatitis infection were severer than those without. HCV-only or HCV plus HBV-infected subjects seemed to be significantly severer than HBV-only infected subjects.

**Table 1:** Clinical characteristics of enrolled 180359 participants.

	Total N = 180359	HBV N = 18726 (10.4%)	HCV N = 13428 (7.4%)	B+C N = 1337 (0.7%)	NBNC N = 146868 (81.5%)	P-value
Age(year), mean±S.D	58.5±11.6	55.6±10.6	63.5±10.7	60.5±10.1	58.5±11.6	<0.001
Male sex, n (%)	73556 (40.8)	8644 (46.2)	4720 (35.2)	566 (42.3)	59626 (40.6)	<0.001
AST(IU/L) mean±S.D	26.2±13.5	28.0±14.2	38.6±23.3	38.5±22.4	24.7±11.1	<0.001
ALT(IU/L) mean±S.D	27.1±21.4	30.4±24.1	43.4±36.5	43.8±35.4	25.1±18.0	<0.001
Glucose(mg/dL) mean±S.D	100.0±38.5	98.6±36.4	102.3±41.8	104.2±47.2	100.0±38.3	<0.001
Cholesterol(mg/dL) mean±S.D	208.8±40.0	201.6±37.6	192.7±39.5	194.0±38.4	211.3±39.9	<0.001
Triglyceride(mg/dL) mean±S.D	134.6±101.9	119.8±89.7	113.7±72.9	109.5±75.6	138.6±105.3	<0.001
AFP(ng/mL) mean±S.D	13.0±2415.1	32.0±3023.3	13.5±452.8	6.2±38.3	10.6±2445.1	0.726
Platelet(10^9^/L) mean±S.D	228.9±62.5	214.0±59.4	197.5±60.7	192.2±55.3	234.0±61.9	<0.001
APRI, mean±S.D	0.32±0.22	0.36±0.25	0.56±0.42	0.57±0.41	0.29±0.16	<0.001
FIB-4, mean±S.D	1.49±0.81	1.53±0.83	2.20±1.16	2.11±1.11	1.41±0.72	<0.001
APRI≥0.7, n (%)	8373 (4.6)	1289 (6.8)	3249 (24.1)	318 (23.7)	3517 (2.4)	<0.001
FIB-4≥3.5, n (%)	5323 (2.9)	629 (3.3)	1831 (13.6)	158 (11.8)	2704 (1.8)	<0.001
DM, n (%)	44919 (24.9)	4204 (22.4)	3638 (27.1)	383 (28.6)	36694 (25)	<0.001
Hypertension, n (%)	53244 (29.5)	4936 (26.3)	4488 (33.4)	443 (33.1)	43377 (29.5)	<0.001

Abbrevations: AFP:alpha-fetoprotein; ALT:Alanine transaminase; APRI:Aspartate transaminase-platelet ratio index; AST:Aspartate transaminase; B+C:hepatitis B virus and hepatitis C virus; DM:diabetes mellitus; FIB-4:fibrosis 4; HBV:hepatitis B virus; HCV:hepatitis C virus; NBNC: non-hepatitis B virus and non-hepatitis C virus

#### The distributions of APRI / FIB-4 by viral etiology

**Fig 1:**
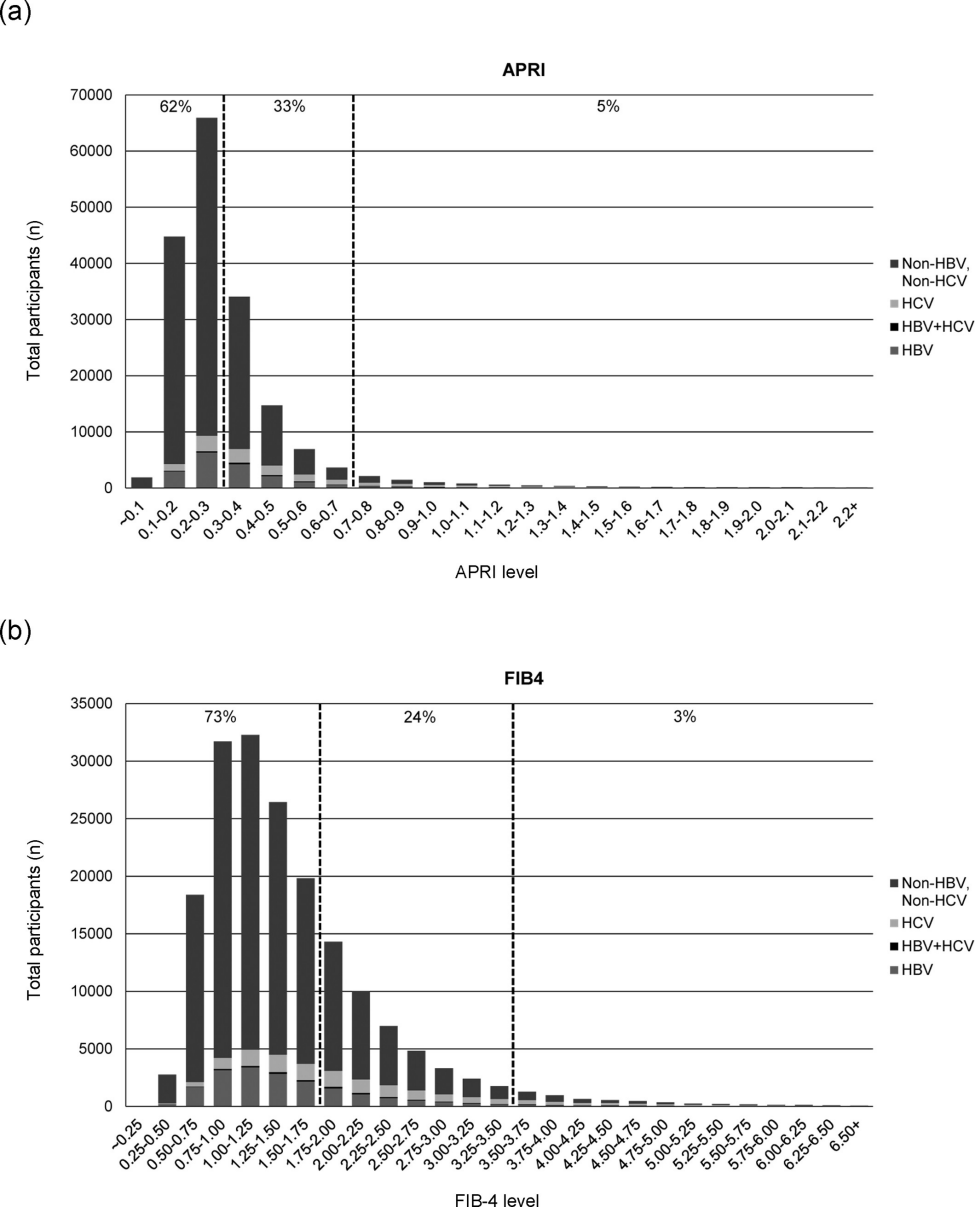
(a). The distribution of APRI by different viral etiology 1(b). The distribution of FIB-4 by different viral etiology.

Fig 1 shows the distributions of APRI and FIB-4 based on different viral etiologies. Approximately 62% of the general population had a APRI level less than 0.3, while only 5% of subjects had APRI higher than 0.7 (Fig 1A). The prevalence of virus hepatitis significantly increased from 29.2%, 46.3%, 58%, 64.5% to 70.4% with the elevation of APRI cut-offs from 0.3, 0.5, 0.7, 0.9 to 1.1 (p<0.001 for the increasing trend) (Table 2). Further focusing on HCV-infected subjects, the prevalence also increased from 13.9%, 28.1%, 38.8%, 45.2%, to 49.9% with the elevation of APRI cut-offs (p<0.001 for the increasing trend). Regarding the prevalence of HBV infection, the level was maintained in the range about 14% to 16% in different cut-offs of APRI. Approximately 73% of the general population had an FIB-4 level less than 1.75, while only 3% subjects had FIB-4 higher than 3.5 (Fig 1B). Like APRI, using FIB-4 to predict the prevalence of chronic virus hepatitis, the rate also significantly increased from 28.2%, 39.1%, 49.2% to 55.5% with the elevation of FIB-4 from 1.75, 2.75, 3.5 to 4.25 (p<0.001 for the increasing trend) (Table 2). In HCV infection, the prevalence increased from 15.8%, 26.4%, 34.4% to 39.7% with the elevation of cut-offs (p<0.001 for the increasing trend). Likewise, the prevalence of HBV infection was around 10.8% to12.6% and was not related to the cut-offs of FIB-4.

**Table 2:** Distibutions of virus hepatitis in enrolled participants based on different cut-offs of APRI and FIB-4.

	APRI ≥0.3	≥0.5	≥0.7	≥0.9	≥1.1	≥1.3
Total	67744	18956	8373	4806	2962	1875
HBV (%)	(13.7)	(15.4)	(15.4)	(14.7)	(15.6)	(16)
HCV (%)	(13.9)	(28.1)	(38.8)	(45.2)	(49.9)	(52.9)
B+C (%)	(1.5)	(2.8)	(3.8)	(4.6)	(4.9)	(5.1)
NBNC (%)	(70.8)	(53.7)	(42)	(35.5)	(29.6)	(26.1)
	FIB-4 ≥1.75	≥2.75	≥3.5	≥4.25		
Total	48914	12814	5323	2416		
HBV (%)	(10.8)	(11.2)	(11.8)	(12.6)		
HCV (%)	(15.8)	(26.4)	(34.4)	(38.7)		
B+C (%)	(1.5)	(2.3)	(2.9)	(3.1)		
NBNC (%)	(71.9)	(60.1)	(50.8)	(44.5)		
	APRI <0.7 + FIB-4 <3.5	APRI <0.7 + FIB-4 ≥3.5	APRI ≥0.7 + FIB-4 <3.5	APRI ≥0.7 + FIB-4 ≥3.5		
Total	169907	2079	5129	3244		
HBV (%)	(10.2)	(8.8)	(16.4)	(13.8)		
HCV (%)	(5.8)	(14.2)	(33.5)	(47.3)		
B+C (%)	(0.6)	(1.1)	(3.6)	(4.1)		
NBNC (%)	(83.4)	(75.9)	(46.6)	(34.7)		

Abbrevations: APRI:Aspartate transaminase-platelet ratio index; B+C:hepatitis B virus and hepatitis C virus; FIB-4:fibrosis 4; HBV:hepatitis B virus; HCV:hepatitis C virus; NBNC: non-hepatitis B virus and non-hepatitis C virus.

When the prevalence of virus hepatitis was just over or very close to 50%, the cut-off of APRI and FIB-4 was 0.7 and 3.5 repectively. Hence, we further divided all participants into four subgroups according to APRI-0.7 and FIB-4-3.5. The proportion of subjects with APRI<0.7+FIB-4<3.5, APRI<0.7+FIB-4≥3.5, APRI≥0.7+FIB-4<3.5 and APRI≥0.7+FIB-4≥3.5, was 94.2%, 1.2%, 2.8% and 1.8% respectively. The prevalence of HBV and HCV was 8.8% and 14.2% in APRI<0.7+FIB-4≥3.5, 16.4% and 33.5% in APRI≥0.7+FIB-4<3.5, as well as 13.8% and 47.3% in APRI≥0.7+FIB-4≥3.5 respectively (p<0.001).

#### The correlation of APRI / FIB-4 and HCC and viral etiology in townships

**Fig 2:**
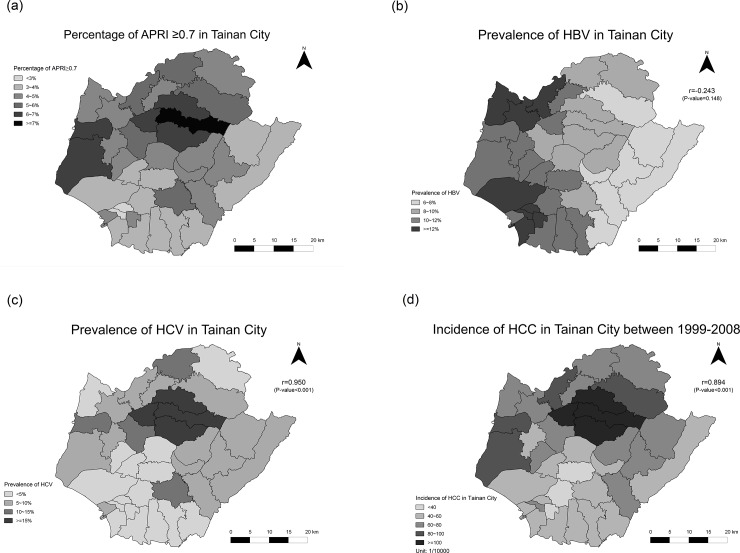
(a). The percentage of 31 townships with APRI ≥ 0.7 in Tainan 2(b). The correlation of APRI ≥0.7 and HBV prevalence in Tainan 2(c). The correlation of APRI ≥0.7 and HCV prevalence in Tainan 2(d). The correlation of APRI ≥0.7 and HCC incidence in Tainan.

**Fig 3:**
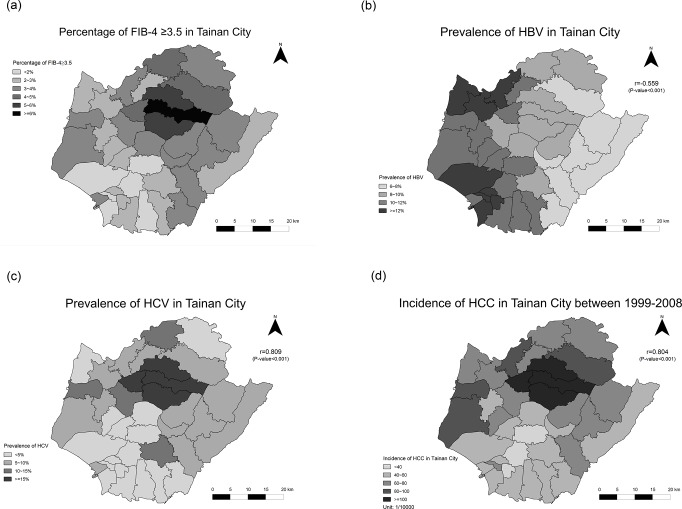
(a). The percentage of 31 townships with FIB-4 ≥ 3.5 in Tainan 3(b). The correlation of FIB-4 ≥3.5 and HBV prevalence in Tainan 3(c).The correlation of FIB-4 ≥3.5 and HCV prevalence in Tainan 3(d). The correlation of FIB-4 ≥3.5 and HCC incidence in Tainan.

Fig 2A shows the percentage of 31 townships with APRI ≥ 0.7 in Tainan. The correlation between the prevalence of APRI ≥ 0.7 and HBV infection was low in township level (Fig 2B). But APRI ≥ 0.7 was highly correlated with the prevalence of HCV infection (r = 0.95, p<0.001) (Fig 2C) and the decade incidence of HCC (r = 0.894, p<0.001) from 1999 to 2008 (Fig 2D). Fig 3A shows the percentage of 31 townships with FIB-4 ≥ 3.5 in Tainan. Likewise, FIB-4 ≥ 3.5 was not correlated to HBV infection (Fig 3B). But FIB-4 ≥ 3.5 also showed a high correlation with the prevalence of HCV infection (r = 0.809, p<0.001) (Fig 3C) and the incidence of HCC (r = 0.804, p<0.001) (Fig 3D).

## Discussion

This is the first study to elucidate the association of APRI, FIB-4 and viral hepatitis in a large-scale community screening. Based on a large-scale database of over 180,000 subjects in the aged population, we obtained the association of APRI / FIB-4 and HBV infection / HCV infection. As we know, the progression of chronic liver disease, LC and its main complication HCC, accounts for the majority of liver-related mortality in the world [20]. CHB and CHC are the two most frequent chronic liver diseases. In the recent decade, the development of potent anti-viral drugs of HBV and HCV has improved the control of chronic liver disease. The prevalence of HBV infection in Taiwan is approximately 15% and has continued to decrease gradually after the offer of the HBV vaccine for newborns since 1984 [3,21]. Regarding HCV prevalence in Taiwan, it is approximately 4% and it is higher in Southern Taiwan than in Northern Taiwan [6]. Although public education of virus hepatitis has been promoted by government or non-government foundations in Taiwan for years, many HBV- or HCV-infected patients are still unaware of the severity of liver disease, especially among elderly patients [22]. Some of the elderly patients don’t even know that they are infected. Large-scale hepatitis screenings in the community might be beneficial to find infected patients who require further hepatitis surveillance. However, to detect serum HBsAg and anti-HCV nationwide is expensive and inefficient. To recognize those highly possibly-infected patients for further hepatitis screening could be more cost-saving. For example, checking the anti-HCV titer in those subjects with APRI ≥1.1 should be reasonable and effective in the community, because the prevalence of HCV infection in this group is possibly close to 50%.

APRI and FIB-4, two non-invasive indexes, have been frequently used to predict liver fibrosis with an acceptable diagnostic performance in different liver diseases including HBV infection and HCV infection [13,14]. Additionally, the two indexes are based on inexpensive laboratory tests and appear well reproducible and easily performed. In this large community-based cohort, 38% of the general population had an APRI level more than 0.3. With the elevation of different cut-offs of APRI from 0.3 to 1.3, the prevalence of chronic virus hepatitis significantly increased from 30% to over 70%. Using FIB-4, 27% of the general population had a FIB-4 level more than 1.75. Likewise, with the elevation of different cut-offs of FIB-4 from 1.75 to 4.25, the prevalence of chronic virus hepatitis increased from 30% to over 50%. We found that the increase of hepatitis prevalence was mainly related to the increase of HCV patients. The prevalence of HBV infection was around 12% to 16% in using APRI and around 10% to 13% in using FIB-4, which was close to the national prevalence of HBV infection in Taiwan. HBsAg spontaneous clearance in chronic HBV infection has been observed in several studies, especially in aged cohorts [23–25]. The annual incidence of HBsAg clearance of these studies was around 1.1%. Our previous study reported that old age was one of the associated factors for HBsAg disappearance, which is a well-known and reasonable factor [25]. Unlike chronic HBV infection, chronic HCV infection in aged cohorts is usually reactive [26]. Our other small-sized community study showed that nearly two-thirds of aged anti-HCV-positive subjects were HCV RNA-positive, and more than half of them had elevated ALT levels [22]. The difference in performance of APRI and FIB-4 in identifying underlying HCV and HBV might be the risk of underlying advanced fibrosis with HCV (chronic hepatitis) relative to HBV (broad phenotype that includes many carriers with indolent disease).

Based on a large-scale database of more than 180,000 subjects, the distribution of chronic virus hepatitis by different cut-offs of APRI and FIB-4 was a good reference to reflect HBV or HCV hepatitis status in other endemic areas. Additionally, in most community-based health screenings, the tests to calculate ARPI and FIB-4 are common and inexpensive. Moreover, those subjects with higher APRI of FIB-4 might have severer liver fibrosis but remain unaware of their liver disease. Hence, we could economically discover those highly possible hepatitis-infected patients requiring further HBsAg and anti-HCV examinations based on different cut-offs of APRI or FIB-4.

In 31 townships of Tainan county, we also found that APRI ≥ 0.7 or FIB-4 ≥ 3.5 was not related to the prevalence of HBV infection at township level. But APRI ≥ 0.7 or FIB-4 ≥ 3.5 was highly correlated with the prevalence of HCV infection (r = 0.95, p<0.001 in APRI; r = 0.809, p<0.001 in FIB-4). This finding was compared with a previous report that the accuracy of APRI and FIB-4 is lower in HBV-infected patients than in HCV-infected patients [14]. Elevated APRI and FIB-4 reflect more advanced liver fibrosis. Because no data of ultrasound or pathology related to real liver fibrosis could be offered in such a large community study, we tried to use the decade incidence of HCC to represent the severe complication of advanced liver fibrosis in Tainan. This study compared the prevalence of APRI ≥ 0.7 or FIB-4 ≥ 3.5 with the decade incidence of HCC in townships of Tainan from 1999 to 2008. The correlation was still significantly high (r = 0.894, p<0.001 in APRI; r = 0.804, p<0.001 in FIB-4). From the township level, this finding hinted that HCC with high APRI or FIB-4 might be more related to HCV infection than HBV infection, which was compatible with a previous study about geographic variations of predominantly HCV associated with males in HCC townships in Taiwan [27].

The study has some limitations. First, APRI and FIB-4 have been proven to predict liver fibrosis with an acceptable diagnostic accuracy, but no data of hepatic fibrosis status identified by abdomen ultrasound or fibroscan could be offered in this large-scale community study. Secondly, our study displayed the distributions of virus hepatitis based on different cut-offs of APRI and FIB-4 in the aged cohort in HBV-and HCV-endemic areas, and we could use this association to discover highly possibly-infected subjects to receive further HBsAg or anti-HCV tests. However, the utility of this association should be cautioned on limited conditions such as use in HBV- or HCV-endemic areas. The association is undetermined in other etiologies. Thirdly, the cause of high APRI or FIB-4 in those NBNC subjects was unclear because detailed information related to liver fibrosis severity such as fatty liver status, body weight or drinking habit, etc., was insufficient. Finally, we did not have longitudinal data on antiviral treatment in this cross-sectional observation study that could impact outcomes.

## Conclusions

In conclusion, the distributions of HCV infection based on different cut-offs of APRI or FIB-4 were clearly elucidated in this large-scale community database. In cautious application of this association, we could identify patient subsets with increased underlying HCV infection. Additionally, APRI or FIB-4 was highly correlated with HCV infection and HCC development, but not HBV infection in the community.
